# Continuous Stress Detection Using Wearable Sensors in Real Life: Algorithmic Programming Contest Case Study

**DOI:** 10.3390/s19081849

**Published:** 2019-04-18

**Authors:** Yekta Said Can, Niaz Chalabianloo, Deniz Ekiz, Cem Ersoy

**Affiliations:** Department of Computer Engineering, Boğaziçi University, Bebek, Istanbul 34342, Turkey; niaz.chalabianloo@boun.edu.tr (N.C.); deniz.ekiz@boun.edu.tr (D.E.); ersoy@boun.edu.tr (C.E.)

**Keywords:** stress recognition, machine learning, wearable sensors, smartwatch, photoplethysmography, electrodermal activity, daily life psychophysiological data, heart rate variability

## Abstract

The negative effects of mental stress on human health has been known for decades. High-level stress must be detected at early stages to prevent these negative effects. After the emergence of wearable devices that could be part of our lives, researchers have started detecting extreme stress of individuals with them during daily routines. Initial experiments were performed in laboratory environments and recently a number of works took a step outside the laboratory environment to the real-life. We developed an automatic stress detection system using physiological signals obtained from unobtrusive smart wearable devices which can be carried during the daily life routines of individuals. This system has modality-specific artifact removal and feature extraction methods for real-life conditions. We further tested our system in a real-life setting with collected physiological data from 21 participants of an algorithmic programming contest for nine days. This event had lectures, contests as well as free time. By using heart activity, skin conductance and accelerometer signals, we successfully discriminated contest stress, relatively higher cognitive load (lecture) and relaxed time activities by using different machine learning methods.

## 1. Introduction

Daily life stress is an important problem of our modern society. It is a growing issue and it has become an unavoidable part of our daily lives. Psychological stress types can be listed as acute and chronic [1]. Acute stress is more prevalent than chronic stress. American Psychological Association noted that the causes of acute stress are pressure from recent past and near future [2]. Athletic challenges, test taking, or anxiety when meeting new people can induce acute stress. On the other hand, long-standing pressures and demands as a result of socioeconomic conditions, difficulties in interpersonal relationships, or an unsatisfying career can trigger chronic stress [2]. If chronic stress is not handled properly, it could result in serious health issues [3]. Since symptoms of acute stress are more apparent than chronic stress symptoms, acute stress is more widely investigated.

After musculoskeletal illnesses, which also could be stress-related in some cases [4], stress is one of the most significant health problems in the world. The effect of stress on human health depends on the stress type. Emotional distress, muscular ache and tension, back pain, headache, heartburn, digestive tract issues, and overarousal can be named as the effects of acute stress [5]. Overarousal can cause heart attacks, arrhythmias, and even sudden death for people with heart conditions [6]. Effects of the chronic stress on human health are akin to those of acute stress however it can damage physical conditions more. Possible causes of the chronic stress can be listed as hypertension and coronary disease [6,7], irritable bowel syndrome, gastroesophageal reflux disease [8], generalized anxiety disorder, and depression [9]. The above-mentioned stress-related diseases also affect the economy by increasing absenteeism, staff turnover [10], presenteeism, and tardiness. These problems decrease the production and increase the work-related costs. Public surveys [11] unveiled that at least half of the European workers are subjected to stress at work. Furthermore, at least half of the lost working days in the business sector are assumed to be caused by work-related stress and psycho-social risks [12].

Researchers found out that stress should be handled when the symptoms first come out to avoid the long-term consequences. In other words, stress must be discovered in early stages to refrain from more damages and impede it from being chronic. The above-mentioned damages of stress on human health and detriments to social life and economy have forced researchers to come up with an automatic stress monitoring scheme which exploits smart wearable devices and advanced affective computing algorithms. This scheme can be applied in automobiles, airplanes, factories, and offices, at job interviews and daily life environments. This scheme can further compute social stress stages during meetings or mutual intercommunication. The ideal scheme should be applicable to daily life, i.e., it should use unobtrusive sensors and devices which users can wear easily in their daily routines.

In this work, we developed an automatic stress level detection scheme that uses physiological signals from wrist-worn devices. Our scheme can also be applied to daily life of individuals. In real-life settings, movements of individuals are unrestricted and artifacts occur because of that. In order for our system to be applicable in these settings, we applied several novel artifact detection and removal strategies. These artifact detection algorithms are developed for specific sensors and their performances are scientifically proven. We further extracted features from heart activity, skin conductance, and accelerometer signals with our tools. From these features, we classified the stress level of an individual by employing machine learning algorithms. To test our system in real-life settings, we collected physiological signals of participants in an algorithmic programming summer camp via smart wrist-worn wearable devices. This camp was composed of lecture, contest, and free time sessions. We collected data for nine days from 21 participants. After the data were collected, we obtained promising results for detecting stress with these wearable devices in real life scenarios. Our work addresses five prominent research issues:

- The comparison of stress detection model performances with different wearable devices;
- The influence of the interpolation, aggregation window sizes and artifact detection threshold percentages;
- Change in the performance of the stress detection scheme with known context labels and the subjective reports as the ground truths;
- The discriminative effect of each sensor modality; and
- The performance of person-specific and general models.

The structure of the rest of the paper is as follows: In Section 2, the related work for stress detection is provided. Real-life data collection problems are addressed in Section 3. In Section 4, our stress detection scheme is explained. Data collection event and our experiment design are presented in Section 5. In Section 6, we present experimental results and discussion. The conclusion of the study and future work are given in Section 7.

## 2. Related Work

The early stress detection research was performed in the laboratory environments, while the current research continues on real-life environments (see Table 1). Electrodermal activity (EDA), heart activity (HR) and accelerometer are the most widely used physiological signals for the detection of stress levels. As shown in Table 1, EDA and HR combination has the best performances in the laboratory environments. Proposals with accuracies higher than 95% use this combination as the physiological signals. Linear Discriminant Analysis (LDA), Support Vector Machine (SVM), k Nearest Neighbors (kNN) and Fuzzy Logic classifiers are the best performing machine learning (ML) algorithms. An 89% accuracy was achieved in four-class stress classification by using EEG signals in [13]. However, current EEG (Electroencephalogram) measuring devices are obtrusive for individuals and they are not applicable to daily life routines.

Almost all of the studies in Table 1 employed a two-class stress level classification. However, the stress up to a certain level might be harmless. After a certain limit, the stress level should be detected and precautions taken. To this end, stress detection resolution must be increased (precision of detected stress levels should be increased) and multi-level stress detection systems with high classification performance must be developed. These schemes should further take advantage of multimodality to increase accuracies as the laboratory research suggest the benefits (see Table 1).

It is recognized that the stress level that subjects endure in this environment is different from real life stress [1]. It is also demonstrated that subjects are reluctant to wear obtrusive instruments for measurement and they are not comfortable with these devices. For these reasons, stress measurement research has taken a step outside the lab with the aim of developing an unobtrusive multi-level stress detection system for daily life. Since smartphones and wearable devices have become an integral part of our lives in our modern society, they are chosen as the instruments for stress detection in daily lives research.

After laboratory environments, stress level detection research has been conducted in restricted and semi-restricted environments such as office, automobile and university campus. Office and workplace are among the environments which increase the stress levels the most. The stress level in the office environment is monitored by using EDA, ECG (Electrocardiogram) and Accelerometer [36,37,38]. Especially in crowded cities, the stress levels of individuals increase in traffic jams. There are a number of studies in the automobile environments in the literature. Most of the studies used DriveDB database [39]. This database consists of ECG, EDA, EMG (Electromyogram) and respiratory sensor data collected from 24 drivers in Boston. Researchers applied machine learning algorithms to this database and EDA-ECG signal combination and SVM-kNN classifiers achieved the best performance [40] in this environment. Campus environments are semi-restricted environments and the most similar environment to unrestricted daily life environment. Therefore, classification performances are lower when compared with restricted laboratory, office and automobile environments. ECG signal and the decision tree classifier have achieved the highest classification accuracy in two-class classification in a campus environment [41]. Most works have only used features extracted from the smartphones [42,43,44]. Smart wearable devices are not used in the campus environment in most of the works.

The stress detection research has taken a step to the unrestricted real life since the ultimate aim is to detect stress levels of individuals in their daily routines. However, researchers should come up with solutions to new problems arise when taking a step outside the laboratory (see Section 3). The stress level recognition performances of real-life schemes are lower than restricted environments and laboratory environments [32] (2016), ref. [45] (2015), ref. [34] (2017) and [35] (2018). The listed works have classification accuracies around 70% and 80%. Low reliability of self-report answers, the unknown context of participants and unrestricted movements of subjects could be the main reasons. Furthermore, the devices used in real-life studies are non-obtrusive but their data quality is not comparable with their laboratory counterparts. There are a lot of smart unobtrusive wearable devices for daily life usage. However, their data quality and effect on stress level detection performance are not investigated thoroughly. Another open research question for real-life studies is the unknown context and low reliability of self-reports. Gjoreski et al. [34] employed activity recognition to increase the knowledge regarding context and improve their recognition performance. The effect of context and questionnaires to the performance of stress recognition systems should be investigated comprehensively. Lastly, to eliminate the negative effects of unconstrained movements, artifact detection and removal algorithms specific for each sensor must be developed and used.

## 3. Preprocessing and Feature Extraction: Problems and Possible Solutions in Real Life

Real-life data collection brings new problems that are not encountered in laboratory data collection. In a lab experiment, methods for data collection are less error-prone and relatively easy. In real life scenarios, new parameters that can cause new research problems are added to the system. For example, the maximum runtime of the devices is limited due to their limited battery. Incorrect placement of devices, loosely worn equipment, charging of instruments, unconstrained movement of subjects and issues with the ground truth collection should be taken into account.

### 3.1. Problems Related to the Movement and Improper Placement of Devices

Today’s off-the-shelf wearable devices provide us with high-quality data standards [46]. However, certain conditions must be satisfied for high-quality data acquisition. Electrodes should be properly placed obeying the instructions of the equipment, wristbands must be tightly worn and body movements should be limited. Otherwise, signals are contaminated by noise, loosely worn devices, and body movements [12]. To remove the noise, some signal processing techniques must be applied. Every problem creates multiple options for researchers. To give an illustration, if a subject wears the device loosely, and for some period the data could not be acquired, the researcher may opt to ignore this time period or interpolate the data. Another example would be the choice of handling data artifacts due to unconstrained movement of a subject in daily life. To clean the data, there are several filters such as Kalman filters, Butterworth low-pass filters, median filters, Wiener filters, and wavelet decomposition [12]. For removing the artifacts, least mean squares, regression analysis, independent component (ICA) and principal component analysis (PCA) could be employed [12].

### 3.2. Data Fusion from Variety of Sensors

To increase the success of stress measurement systems, researchers tend to collect multimodal data. The integration of multimodal data imposes challenges. Synchronization must be employed between different data types by using timestamps. When to integrate the data (before classification or during processing) and missing data from some modalities are other challenges.

### 3.3. Selection of Non-Obtrusive Devices

To collect data during the daily life of individuals, stress measurement devices should be non-obtrusive. People should wear these devices without being uncomfortable in their daily routines, during sleeping, meetings and everyday activities. Obtrusiveness may even lead to extra stress on participants. The ideal system should collect the data without the user even being aware of it.

### 3.4. Limited Runtime Due to Battery

Limited runtime is another significant problem when collecting data from participants in real life. If the maximum runtime of a device is around 3–4 h (such as the case of Samsung Gear S1, S2, and S3), researchers or users have to charge the device several times for a whole day of data collection. This causes gaps in the collected data and increases the amount of effort for recharging and restarting sessions. The imposed challenges on researchers would be increasing the battery lives of devices by reducing power consumption (i.e., disabling some sensors, duty cycling devices, and decreasing brightness).

### 3.5. Ground Truth Collection

In laboratory experiments, researchers know the ground truth such as relaxed, baseline, and stressed because they designed the experiment timeline. However, in real life data collection, to measure the success of stress detection schemes, the ground truth from subjects must be collected. To this end, researchers usually employ some surveys (Perceived Stress Scale, Stress Self-Rating Scale, NASA-TLX, The State-Trait Anxiety Inventory, Self Assessment Manikin and Positive and Negative Affect Schedule questionnaires)periodically during a day. Researchers have to collect the surveys from each participant and redistribute new ones when the time comes. This task can be automated by developing a mobile survey app and collecting answers periodically through pop-up surveys. In our case, we have the context information during the data collection, such as if they are in a lecture, a contest or free time.

## 4. Proposed System Description

In plethysmography, volumetric changes of organs are measured from the skin illuminated by the light emitted from a pulse oximeter PPG [47]. PPG sensors in our devices are used to measure the heart activity by measuring blood flow during the heart’s pumping actions. Heart activity signal is composed of different peaks and valleys. R peak is the most prominent one, which is used to calculate heart rate variability. PPG provides the RR interval by measuring the duration between two consecutive R peaks which can also be called as Interbeat Interval (IBI).

EDA also known as Galvanic Skin Response (GSR), is the change of electrical properties of skin. Under emotional arousal and stress, body sweats and skin conductance increases. EDA is one of the best and widely used discriminative signal along with the heart rate signal for measuring stress [48]. Mean amplitude, standard deviation, minimum and maximum values, RMS, the delay between applied stimuli and response, number of peaks, peak height, rising time, recovery time, the position of maximum and minimum features were used in the literature to measure the stress levels of the user [49].

**Figure 1:**
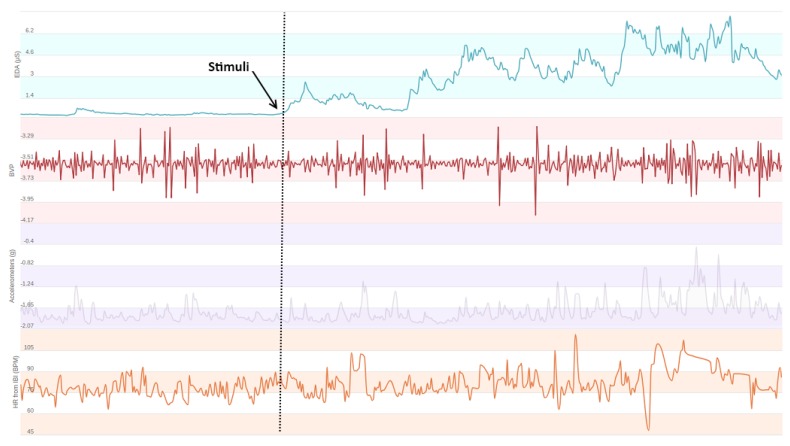
Recorded physiological signals before and after the start of the stimuli. The increase in EDA signal level and number of peaks and irregularities and sudden increases in HRV can be seen in this figure.

**Figure 2:**
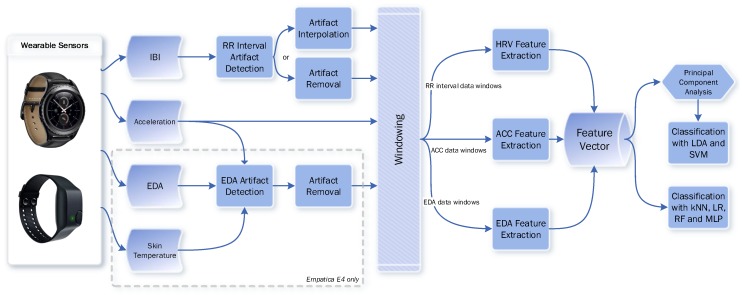
The block diagram of the stress level detection system for Samsung Gear S and S2 and Empatica E4. Since the sensors and platforms are different, please note that EDA and temperature signals are only available for E4.

In this study, we developed a multi-level stress detection system, which employed heart activity data from the PPG sensor, skin conductance data from the EDA sensor and accelerometer and temperature data. Our EDA preprocessing tool uses accelerometer and temperature signals to clean the artifacts in this signal. We further extracted features from the accelerometer sensor but temperature data were not used for feature extraction. The increase in the heart rate and electrodermal activity levels can be seen in Figure 1. Preprocessing and feature extraction tools for each modality were developed. For each sensor, modality-specific tools were applied to eliminate artifacts, cleaning signals and extracting features. After the feature extraction, the most successful machine learning algorithms in the literature were applied to the physiological data for the classification task. Our system is compatible with different smart wrist-worn wearables in spite of the fact that they have different platforms and sensors. System diagrams for Samsung Gear S family devices and Empatica E4 devices are shown in Figure 2. Note that all parameters for artifact detection and preprocessing algorithms are universal and person independent.

### 4.1. Electrodermal Activity Signal Preprocessing and Feature Extraction Tools

#### 4.1.1. Preprocessing and Artifact Removal

**Figure 3:**
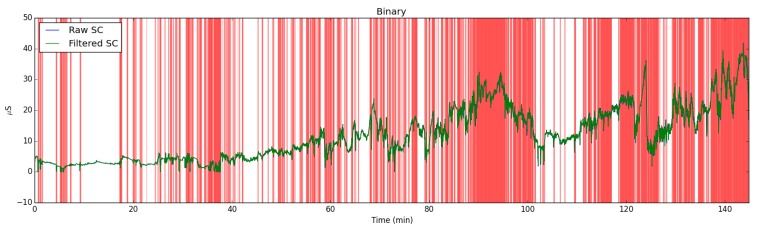
The example filtered EDA signal according to changes in the accelerometer signal. Note that red components were deleted because of the high activity intensity.

**Figure 4:**
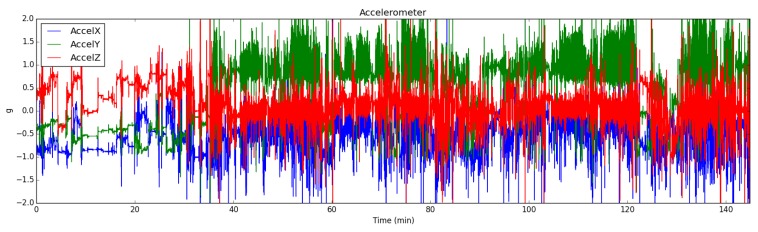
Activity intensity is shown by using the accelerometer sensor X, Y, and Z components corresponding to the example EDA signal in Figure 3 Note that this example was recorded during a highly intensive activity.

Electrodermal Activity signal is affected by increased physical activity and temperature changes. In these situations, obtained signal is contaminated and should be filtered. To this end, we employed the EDA Explorer tool from Taylor et al. [50]. The artifacts in the EDA signal is manually labeled by the experts to train a machine learning model. By applying the SVM (Support Vector Machine) classifier with the accelerometer and temperature data, this tool achieves 95% accuracy on detecting artifacts in the EDA signals (see Figure 3 and Figure 4). We added batch processing feature to this tool. If a data segment is detected as an artifact segment, it is excluded in the feature extraction process. By this way, we eliminated false peaks caused by increased temperature or physical activity when extracting features.

#### 4.1.2. Feature Extraction

**Figure 5:**
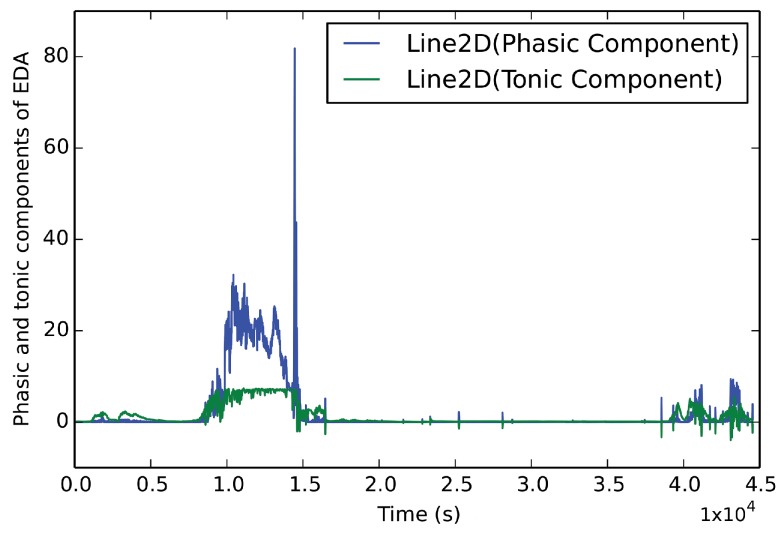
Decomposed EDA Signal from Empatica E4 wristband by applying cvxEDA tool.

After cleaning artifacts from the signals, features were extracted. EDA signal has two components: phasic and tonic. We decomposed the EDA signal by applying the *cvxEDA* tool [51] on the EDA signal, which makes use of a convex optimization approach to decompose the EDA signal. Skin Conductance Level (Tonic) component includes more long-term slow changes, whereas phasic components include faster (event-related) changes. When evaluating the mean, standard deviation, and percentile features, researchers use the tonic component because they do not want to overestimate these long-term changes with event-related fast changes. The phasic part is subtracted and features are calculated. On the other hand, some peak related features such as peaks per 100 s, peak amplitude, and strong peaks (peaks that are more than 1 μSiemens) per 100 s are calculated from the phasic element. An example of a decomposed signal is shown in Figure 5. After that, we extracted seven features from the EDA signal: mean, standard deviation, peak, strong peak, 20th percentile, 80th percentile and quartile deviation (75th percentile–25th percentile).

### 4.2. Heart Activity Signal Preprocessing and Feature Extraction Tools

#### 4.2.1. Preprocessing and Artifact Removal

**Figure 6:**
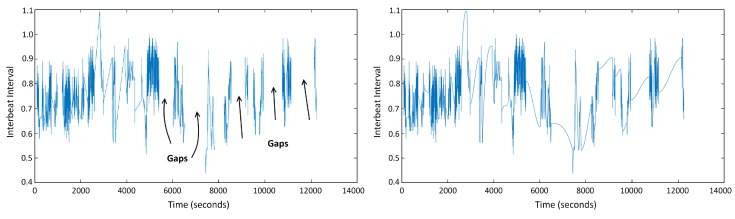
Gaps due to movement and loosely worn wristband from PPG (Photoplethysmography) data (**Left**) are filled with cubic interpolation function (**Right**).

The heart rate activity signal is also sensitive to the movement of the subjects and loosely worn wrist devices. To cope with these problems and clean the artifacts from the signal, our research group developed a preprocessing tool in MATLAB. With this tool, we employed an artifact detection percentage threshold between the data and the local average. In the literature, this threshold is generally set as 20% [38] and we also used this threshold. After we detected the artifacts in the heart activity signal, a user can choose to remove and apply some additional constraints or replace them with shape preserving cubic spline interpolation after removal (see Figure 6). If the artifact data points are removed and not-interpolated, new rules can be set on the remaining healthy data. A minimum amount of consecutive data samples and minimum consecutive time rules can be set to evaluate the remaining segments. These rules are used to exclude interrupted (with holes of removed data) small amount of consecutive data in the feature extraction process. We applied removal with extra exclusion rules and removal and interpolation separately and observed their effect on the performance of our system.

The tool also has a batch processing feature. Length of the local mean, the percentage of artifact detection threshold, minimum consecutive time and data sample constraints can be altered with parameters.

#### 4.2.2. Feature Extraction

For feature extraction, we used MATLAB built-in tools along with Marcus Vollmer’s HRV toolbox [52] along with our preprocessing tool. The employed time domain features are the mean value of the heart rate (Mean HR), the standard deviation of inter-beat interval (IBI), mean value of the inter-beat (RR) intervals (Mean RR), root mean square of successive difference of the RR intervals (RMSSD), the percentage of the number of successive RR intervals varying more than 50 ms from the previous interval (pNN50), the total number of RR intervals divided by the height of the histogram of all RR intervals measured on a scale with bins of 1/128 s (HRV triangular index), and triangular interpolation of RR interval histogram (TINN).

We also applied Fast Fourier Transform (FFT) and Lomb–Scargle periodogram [53] and the following frequency domain features are calculated: low frequency power (LF), high frequency power (HF), very low frequency power (VLF), prevalent low frequency (pLF), prevalent high frequency (pHF), the ratio of LF to HF (LF/HF), (From Lomb–Scargle) LF, HF, and LF/HF. Definitions of these features are given in Table 2.

### 4.3. Accelerometer Processing and Feature Extraction

Body and head movements can be used to detect the emotions and arousal level [54]. The accelerometer sensor records three-axis acceleration with gravity. We employed the accelerometer modality in two ways. Firstly, to detect artifacts in the EDA data, accelerometer data were used along with the temperature data. Secondly, we used these data for feature extraction. The mean value is calculated for each window. The energy of the signal is also calculated with FFT.

### 4.4. Machine Learning Tools

For the classification of the data, we employed the Weka toolkit [55]. For preprocessing of features, we applied numeric to nominal transformation to the class column. Since our dataset is unbalanced in terms of membership of class instances, we added instances from the minority class and removed the samples from the majority class to overcome the class imbalance problem. Therefore, we prevented classifiers from biasing towards the class with more instances. In this study, we evaluated the performance of six well-known classifiers.

1. Principal Component Analysis (PCA) and Linear Discriminant Analysis (LDA)
2. PCA and Support Vector Machine with radial kernel (SVM)
3. K-Nearest Neighbours (*n* = 1) (kNN)
4. Logistic Regression
5. Random Forest (RF with 100 trees)
6. Multilayer Perceptron

Ten-fold cross-validation was applied. A three-class classification system was developed. The parameters of the classifiers were selected from the stress level detection studies in the literature.

## 5. Description of the Data Collection Event: Algorithmic Programming Summer Camp

**Figure 7:**
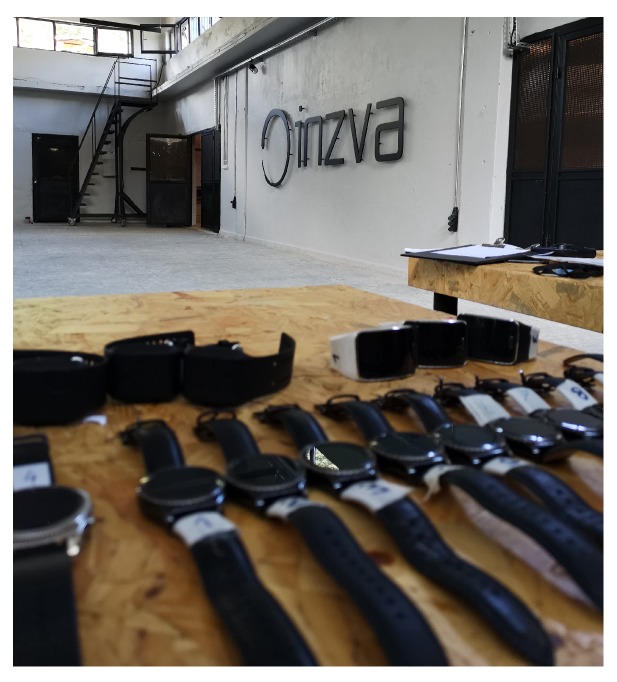
A view of smartwatches and wristbands after data extraction, charged and ready to use.

To test and evaluate our system in real-life settings, we conducted a data collection experiment in the INZVA algorithmic programming contest summer camp, which is organized each year in Istanbul, Turkey [56]. This event is similar to the International Collegiate Programming Contest (ICPC) [57]. A photograph from the data collection setup is shown in Figure 7. Algorithmic programming camp is designed for high-school and university students to improve their programming skills and this contest will induce stress on the participating students.

The algorithmic programming contest is conducted in three levels, expert, advanced and foundation. Eighty-four students with different levels of expertise gathered to participate in this algorithmic programming contest. Algorithmic programming contest camp was held for nine days. The physiological signal and questionnaire data were collected from the 21 participants at the foundation level. Of these 21 people, 18 were men and 3 female and the average age of the attendees was 20.

**Figure 8:**
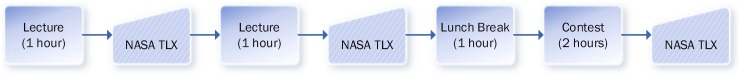
The daily schedule and data collection procedure during the algorithmic programming contest.

There were three types of sessions such as the training, the contest and the free day. The program was scheduled to be held from 10:00 to 17:00 for the whole nine days. The data collection schedule presented in Figure 8 was repeated for the first eight days. In those days, attendees had training classes with professors from the field of computer science and computer engineering from high-ranking universities in Turkey for two hours. The participants entered daily problem-solving contests in which the questions were derived from the same days of training lectures. In all sessions, raw NASA-TLX questionnaires were collected from the users. The frustration question of this questionnaire was used to measure the perceived stress levels of the individuals. On the closing day, the participants entered the final contest and they should solve challenging questions, which were asked from all the topics covered in the total eight days of training. Participants had to solve challenging questions within a time limit and the scores of the participants were projected on a wall, which created extra stress for the participants. To collect more points, participants should solve more questions in a shorter time period than their opponents. As a result, the mental demand as well as the temporal demand was increased, which encouraged the participants to gain more points in a shorter time and to achieve a higher position in the final ranking. When a participant solved a question, a balloon was attached to his/her table.

### 5.1. Data Collection Procedure

We informed the volunteer participants about the purpose and the procedure of the study. The data collection procedure and all the interventions in this research fully meet the 1964 Declaration of Helsinki [58] and before any data acquisition, all participants volunteering to take part in the study first signed the informed consent documents.

Twenty-one foundation level subjects were selected considering that they would attend the entire week and with approximately the same conditions. Tutorials and further guidelines were presented to all of them concerning how to use the devices and how to fill in the questionnaires. Three study investigators checked if the devices were worn properly and running correctly. For data extraction, collecting the forms and battery recharge procedures, which were being administered by our team, a schedule was set up and participants followed this schedule regularly.

A unique number was assigned to each participant and to each device during the study. Study investigators ensured that the participants wear the device with the correct number. After the data collection, the relations between the participant names and the numbers are anonymized.

### 5.2. Ethics

The procedure of the methodology used in this study was approved by the Institutional Review Board for Research with Human Subjects of Boğaziçi University with the approval number 2018/16. Prior to the data acquisition, each participant received a consent form, which explains the experimental procedure and its benefits and implications to both the society and the subject. The procedure was also explained vocally to the subject. The data collection procedure and all of the interventions in this research fully meet the 1964 Declaration of Helsinki [58]. All of the data are stored anonymously.

### 5.3. Types of Wearable Devices Used for Data Acquisition

Two Samsung Gear S1, ten Samsung Gear S2, four Samsung Gear S3 smartwatches and four Empatica E4 wristbands were used to gather data in this event. The maximum runtime of the devices when they are fully charged varies. While Empatica E4s can collect data for over 48 h, Samsung smartwatches can collect data for at most 4 h when all the sensors are active. All these devices are off-the-shelf and they provided us with the ability to access the raw data. Empatica E4 is solely built for research and it provides the software for accessing data; however, we had to develop an application for data acquisition for Samsung devices that allows the selection of sensors to be used for data collection. However, for this research, we gathered data from all sensors. While data collected from Samsung devices were collected directly by Wi-Fi, Empatica E4 data were first sent to the cloud.

While Empatica E4 devices have four sensors, namely 3D accelerometer (ACC), photoplethysmography (PPG), electrodermal activity (EDA) and the skin temperature (ST), Samsung Gear watches lack the EDA sensor but instead are equipped with Gyro and Barometer sensors. In this study, we used PPG, EDA and ACC sensors.

## 6. Discussion of Experimental Results

We developed a three-class stress detection system. The system can differentiate the stress level of the free day, lecture and contest sessions. It can further differentiate three levels of perceived stress (see Section 6.5). Besides from Section 6.5, we used the context label as the ground truth and we called the measured stress as physiological stress. Free day is enumerated as 0, lecture is assigned 1 and contest is assigned 2 labels. We assumed that the stress levels of most of the subjects would be higher in contest, medium in lecture and lower in the free time with this context labels. In Section 6.5, the frustration scale of raw NASA-TLX is further used as a ground truth and performance of the system is compared under these two conditions. With the latter ground truth, perceived stress level of individuals from self-reports was measured.

### 6.1. Effect of Different Physiological Modalities

Multi-modality of any stress detection scheme is proven to improve the accuracy and performance of the systems. However, the effects of each modality and their combinations are different when the performance is taken into consideration. We examined the effect of each modality. The heart activity alone, heart activity and accelerometer combination, heart activity and electrodermal activity combination and all of the modalities together were investigated, as presented in Table 3. We achieved the highest stress–activity level detection accuracies when MLP was applied to the features from all the modalities. On the other hand, heart activity and electrodermal activity combination for stress level detection achieved the best results with the logistic regression classifier.

We divided the performance evaluation into two categories. In the first category, heart activity and electrodermal activity signals were used to detect physiological stress levels. In the second category, we added accelerometer signal to these signals. With the addition of the data from the accelerometer sensor, information regarding the activity and the context of individuals were also evaluated in this category. To this extent, we called this category as “Stress with Context”. In our case, since we knew the context for all times, adding of accelerometer features to the feature vector might be trivial and these features increased the performance of our system. However, we added these features to show that context information is crucial in daily life studies; when it is completely unknown, adding them will also increase the performance of those systems. When we combined HR and EDA signals, the accuracy was higher than either signal alone in almost all cases (in RF, it was equal to HR). We can infer from that using multiple modalities increases the performance of the stress level detection schemes.

The detailed stress with context detection accuracy results, f-measure, precision and recall values are presented in Table 4, Table 5 and Table 6. Table 4 presents the classification accuracy results obtained from HR, EDA and ACC signals of the Empatica E4 device. The multilayer perceptron algorithm achieved the best classification accuracy of 92.15%.

In Table 5, we leave the EDA signal collected from Empatica E4 out, since Samsung Galaxy Gear devices do not have EDA sensors. In Table 6, we demonstrate the results from the data collected from 18 participants for 32 h (nine days) by using four Empatica E4 and 14 Samsung Gear devices (All Devices). Note that we also collected data with the Samsung Gear S3 classic smartwatch from three participants. However, we did not use these data since office Samsung SDK no longer provides the RR interval of raw data. The Multilayer Perceptron algorithm achieved the best result (92.19%) from HR and ACC signals collected using Empatica E4, whereas the Random Forest algorithm gave the best classification accuracy (88.26%) with the HR and ACC data collected from all devices.

### 6.2. Effect of Device Type

We compared the effect of using Empatica E4 and Samsung Gear S-S2 devices (Combination of Samsung Gear S and S2 devices) relative to each other. Samsung Gear S-S2 devices are a commercial type, relatively cheaper smartwatches. On the other hand, Empatica E4 is a more precise, relatively more expensive research device. We compared the classification accuracies and data quality on both of these devices.

**Figure 9:**
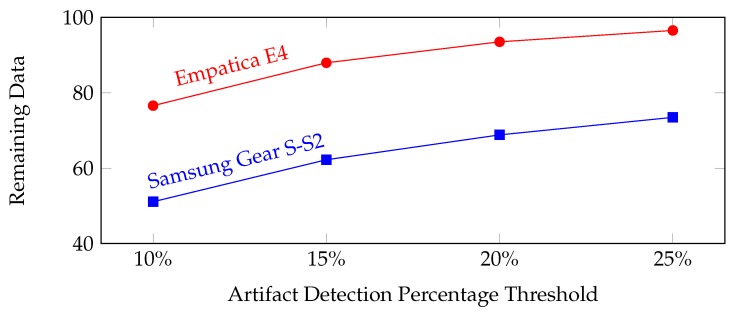
Percentage of the remaining data (for both device types) after the artifacts are removed versus different percentage thresholds of artifact detection.

In the literature, RR intervals that differ more than 20% of the local average are removed [38]. This is called the artifact detection with a percentage threshold. We changed the value of this threshold from 10% to 25% and observed the amount of remaining clean data. As shown in Figure 9, Empatica E4 devices have approximately 25% more remaining data for all of the different artifact detection percentage thresholds. We deduced that the quality of RR intervals of Empatica E4 devices is higher than those of the Samsung Gear S-S2 devices. We further investigated the effect of data quality on stress level classification accuracies. We observed that classification accuracies obtained from the data collected with Empatica E4 were higher than those from Samsung devices with all classifiers, as shown in Table 7 and Table 8. From these results, we can observe that the data quality has a significant effect on the stress level classification accuracy.

### 6.3. Effect of Artifact Detection Percentage Threshold, Interpolation and Aggregation Window on Accuracy

Physiological signals are sensitive to the movements of the subjects. Especially the quality of the heart rate data declines very drastically in the case of intense physical activities. We applied a few preprocessing techniques and filters to remove the contamination of the heart rate data. We investigated the effects of artifact detection percentage threshold, interpolation and the aggregation window length. Artifact detection percentage threshold is the minimum percentage difference between a data point and the local average to evaluate the data point as an artifact. If the value of the artifact correction percentage threshold increases, the filter loosens, i.e., the number of detected artifacts decreases. Furthermore, the aggregation window is the data segment in which features are extracted and averaged for the whole session to get the features of the session.

We applied artifact correction percentage thresholds from 10% to 25% and investigated the stress level classification accuracies, as shown in Table 9. We were unable to observe a pattern when we applied different classifiers and changed the artifact detection percentage thresholds. We can infer that changing this threshold does not have a clear effect on classification accuracy.

We further examined the effect of aggregation window on the stress level classification accuracy (see Table 10). We changed the length of the aggregation window from 2 min to 20 min. We observed that the behavior changed for each ML algorithm. Researchers should consider the ML algorithm and its performance of different aggregation window sizes when deciding the optimum window length. Gjoreski et al. found that the aggregation window lengths between 10 min and 17.5 min have better accuracy in general [34], which is similar to our results.

As mentioned above, we provide a selector in our heart rate preprocessing tool that decides whether to interpolate the removed artifacts or remove and apply some minimum consecutive rules. Minimum consecutive rules could be either the minimum required number of samples or the time interval for a segment to extract features. We further investigated the effect of removal and interpolation to the classification accuracies. In Table 11, we can see that applying interpolation achieved higher performance than filtering for some machine learning methods (removal and minimum consecutive filter) and lower results for other algorithms. This decision depends on the applied ML method.

### 6.4. Person Independent and Dependent Models

We developed two different stress detection systems. The first one is the general (person-independent) model. In this model, the collected data from all of the participants are divided into training and test segments without considering the participant labels. By employing 10-fold cross-validation, the accuracy of the system is determined independently from any individual’s data. The second model is the person dependent model. In this model, the data collected from different participants are divided. After this division, training and test partitions are divided for each person and models are developed for each participant. The classification accuracy is calculated for each individual and an average of all accuracies of the participants is presented. Since everyone has a particular stress behavior and person-specific models take only individuals data into consideration when developing models, these models are expected to have a higher performance. We present the accuracy results in Table 12. We observed that person specific stress detection models had higher classification accuracies than general models, as expected. Furthermore, we achieved the highest classification accuracy on person-specific models with Empatica E4 devices when the Random Forest algorithm was applied (97.92%) to features from all signals. With all algorithms, HR, EDA and ACC signal combination with Empatica E4 devices had higher accuracy than with all devices in person-specific models. These results demonstrate that stress level detection schemes should give more weight to the individual’s data than data from other people when building models.

### 6.5. Measuring the Perceived and Physiological Stress

As can be seen in the literature, in the same context, the perceived stress and physiological stress of individuals can be different. We investigated the effect of two different ground truth collection methods in this subsection. The first one is the known context as the ground truth. In our case, the contest context was assumed to induce stress, the lecture context was assumed to gives some cognitive load and a lower amount of stress and free time was assumed to be relaxed sessions. In this method, the ground truth is enumerated from the known context as: free day, 1; lecture, 2; and contest, 3. When we examined the physiological signals, we could differentiate these three levels with high classification accuracies (see Table 13). Perceived stress of individuals was also measured. We asked the following question to the participants to learn their stress level:

How irritated, stressed, and annoyed versus content, relaxed, and complacent did you feel during the task?

The answers were on a scale of 0–100 with five-point increments. We determined the stress level as 1 if the answer was between 0 and 30. Stress level was assigned to 2 if the answer was 35–75. The highest stress level (3) was assigned if the answer was at least 80.

When we look at the classification accuracies of the perceived stress level, for all ML algorithms and signal combinations, they were lower than the corresponding physiological stress level. This is because the perceived stress is subjective, depending on the individual. The survey answers were also prone to error and this might be another reason for the decrease in the stress level detection accuracies. The correlation between the known context and perceived stress labels was computed to be 0.356, which is a moderate relation. The relation between the perceived stress and physiological stress is not investigated thoroughly in the literature. Development of personal perceived stress level models and filtering out the outlier survey answers might increase the performance of the classifiers.

## 7. Conclusions

We developed a stress detection scheme to be used in real life. Since our system employs unobtrusive wearable devices, it can easily be used in the daily life of individuals. It can track the stress levels in real-time and intervene if an extreme level of stress is detected. After the detection, some stress management methods can also be offered to alleviate the high level of stress. We collected data from participants of an algorithmic programming contest to evaluate the performance of our system. We obtained labeled sessions for 21 subjects for nine days. We described the difficulties of real-life data collection, which do not occur in laboratory environments. After describing our algorithms, we presented the results. For three-class stress level detection, we obtained 90.40% accuracy by using Empatica E4 devices with high data quality, whereas the accuracy with Samsung S devices was 84.67%. We can deduce from the results that the data quality of the devices increases the stress level classification accuracies. When compared with other real-life studies in Table 1, our system has higher accuracies even for the results obtained with the three-class classification. After examining the effect of different preprocessing methods and parameters, we can infer that their effect depends on the chosen ML algorithm. Researchers should select these methods by considering their performance with selected ML algorithms. Furthermore, person-specific models have always higher classification accuracies than general models. We achieved maximum 97.92% accuracy for three-level stress detection with our person-specific models. On the other hand, we obtained a maximum 88.20% classification accuracy with our general models. When physiological data from each person are sufficient for developing person-specific models, they should be applied. Otherwise, people should be clustered according to their stress behaviors and models can be developed for clusters to increase the performance of general models. The best performing classifiers were the Random Forest and the Multilayer Perceptron algorithms. These classifiers outperformed other algorithms in most cases. Another significant finding is that the combination of modalities increases the performance of our system. When we combined heart activity with electrodermal activity, we obtained 92.15% maximum three-level classification accuracy, whereas this was 86.27% when these modalities were used separately. Finally, we observed that the perceived stress level classification results in lower accuracies than physiological stress level classification. There were up to 15% decrease when compared with physiological stress level classification accuracies. The possible causes of this decrease could be listed as subjectivity and fallibility of self-report answers and the possible difference of physiological and psychological responses of individuals to the same stressor. As a future work, we plan to record data with an increased number of high-quality Empatica E4 devices. We further plan to develop personalized perceived stress level models from ground truth surveys and remove outlier answers to increase the perceived stress level classification accuracies.

## Figures and Tables

**Table 1:** Stress detection experiments in controlled laboratory environments.

Article	Stress Signal	Stress Test	Method	# of Classes	Accuracy %	Applicable in Daily Life?
[14] (2012)	HRV	Stress in the traffic	Minimum Distance Classifier	3 (Low, Medium, High)	90	Yes
[15] (2011)	EDA, PPG	Hyperventilation and Talk Prep	Fuzzy Logic	2 (S, R)	99	Yes
[16] (2013)	Speech	TSST	SVM	2 (S,R)	72	Yes
[17] (2011)	ECG, EMG, EDA	Arithmetic, Puzzle, Memory Tasks	Bayes, kNN, LSD	2 (S, R)	80	Yes
[18] (2016)	PPG, EDA, Respiration, Thermal Cam	Lie Detection	DecisionTree	2 (S, R)	73	Yes
[13] (2016)	EEG	Arithmetic Task	SVM	4 (Neutral, Medium, Low, High)	89	No
[19] (2015)	Body Movements	Arithmetic Task	SVM	2 (S, R)	77	No
[20] (2016)	Body Movements, EMG, EDA, Respiration	Arithmetic Task	SVM	2 (Stress, Relax)	85	No
[21] (2017)	Facial Cues	Social Exposure and Stressful Media (IAPS)	kNN, SVM, Naive Bayes	3 (Neutral, Relax, Stressed)	91.68	No
[22] (2014)	Pupil Diameter	IAPS	DecisionTree	2 (Stress, Relaxed)	90	No
[23] (2017)	EDA, PPG, Speech, Accelerometer	TSST	Adaboost	2 (Stress, Relax)	94	Yes
[24] (2015)	EDA, Accelerometer, Bluetooth	—	Logistic Regression	2 (Stressed, Unstressed)	91	Yes
[25] (2012)	Temperature, Heat Flux, EDA, Respiration, Accelerometer	Arithmetic Task, Cold Pressor and loud Sounds	Naive Bayes	2 (Stress, Relaxed)	82	No
[26] (2017)	ECG, GSR, Respiration, Blood Pressure, Blood Oximeter	Ice test and IAPS	SVM, kNN	2 (Stressed, Relax)	95.8	Yes
[27] (2014)	EEG, ECG, EMG, EOG	Mental and Memory Task	ANN	3 (Relaxed, Mental, Fatigue)	80	No
[28] (2015)	Facial Blood Flow	SCWT	Multiple Regression	2 (S, R)	88.6	No
[29] (2015)	EDA	Fail Scenarios	LDA	2 (S, R)	98.88	Yes
[30] (2016)	Human Gaze, Mouse Click	Arithmetic Task	Random Forest	2 (S, R)	66	No
[31] (2018)	BioRadar	Mental Arithmetic Task	Multilayer Perceptron	2 (S,R)	0.94	No
[32] (2016)	Mobile Application Usage Pattern-Physical Activity-Light Sensor-Screen Events	Real Life	SVM, ANN, kNN	2 (S, R)	70	Yes
[33] (2016)	BVP-Skin Temperature-EDA-RR-Heart Rate (Without Context Info)	Real Life	Random Forest	2 (S, R)	76	Yes
[34] (2017)	HR-IBI-HRV-EDA- Temperature	Real Life	Weka Toolkit	2 class (S, R)	70	Yes
[35] (2018)	Phone usage data for different application categories	Real Life	HMM with MPM	2 (S, R)	68	Yes

**Table 2:** Heart rate variability features and their definitions.

Feature	Description
Mean RR	Mean value of the inter-beat (RR) intervals
STD RR	Standard deviation of the inter-beat interval
RMSSD	Root mean square of successive difference of the RR intervals
pNN50	Percentage of the number of successive RR intervals varying more than 50 ms from the previous interval
HRV triangular index	Total number of RR intervals divided by the height of the histogram of all RR intervals measured on a scale with bins of 1/128 s
TINN	Triangular interpolation of RR interval histogram
LF	Power in low-frequency band (0.04–0.15 Hz)
HF	Power in high-frequency band (0.15–0.4 Hz)
LF/HF	Ratio of LF-to-HF
pLF	Prevalent low-frequency oscillation of heart rate
pHF	Prevalent high-frequency oscillation of heart rate
VLF	Power in very low-frequency band (0.00–0.04 Hz)
SDSD	Related standard deviation of successive RR interval differences

**Table 3:** Stress detection accuracies with different ML algorithms: three-class classification. On the left side, stress recognition results that only used HR and EDA signals are presented. On the right side, context information with accelerometer data is also added. The highest accuracy in every column is emphasized with bold.

Algorithm	Stress Only			Stress with Context		
	HR	EDA	HR + EDA	HR + EDA + ACC	HR + ACC	EDA + ACC
PCA + LDA	49.01	52.94	62.70	82.35	72.50	80.39
PCA + SVM (radial)	80.39	62.74	84.31	82.35	86.27	80.39
kNN	82.35	**84.31**	86.27	80.39	84.31	80.39
Logistic Regression	84.21	60.78	**92.15**	90.19	86.27	78.43
Random Forest	**86.27**	80.39	86.27	86.27	**90.19**	**84.31**
Multilayer Perception	**86.27**	68.62	90.19	**92.15**	**90.19**	82.35

**Table 4:** Stress with context classification accuracy, f-Measure, precision and recall values with different ML algorithms: three-class. HR + EDA +ACC for Empatica E4.

Algorithm	HR + EDA + ACC (Empatica E4)			
	Accuracy	f-Measure	Precision	Recall
PCA + LDA	82.35	82.20	82.60	82.40
PCA + SVM (radial)	82.35	82.50	83.30	82.40
kNN	80.39	80.40	80.80	80.40
Logistic Regression	90.19	90.10	90.20	90.20
Random Forest	86.27	86.20	86.20	86.30
Multilayer Perceptron	**92.15**	**92.20**	**92.30**	**92.20**

**Table 5:** Stress with context classification accuracy, f-Measure, precision and recall values with different ML algorithms: three-class. HR +ACC for Empatica E4.

Algorithm	HR + ACC (Empatica E4)			
	Accuracy	f-Measure	Precision	Recall
PCA + LDA	72.54	71.60	71.80	72.5
PCA + SVM (radial)	86.27	86.20	86.90	86.30
kNN	84.31	84.10	84.60	84.30
Logistic Regression	86.27	86.20	86.90	86.30
Random Forest	88.25	88.00	88.10	88.20
Multilayer Perceptron	**92.19**	**90.30**	**91.40**	**90.20**

**Table 6:** Stress with context classification accuracy, f-Measure, precision and recall values with different ML algorithms: three-class. HR +ACC for all devices.

Algorithm	HR + ACC (All Devices)			
	Accuracy	f-Measure	Precision	Recall
PCA + LDA	59.12	59.80	60.10	59.60
PCA + SVM (radial)	76.99	77.10	77.30	77.00
kNN	87.32	87.20	87.30	87.30
Logistic Regression	65.25	65.00	65.00	65.30
Random Forest	**88.26**	**88.20**	**88.20**	**88.30**
Multilayer Perceptron	83.09	83.00	83.20	83.10

**Table 7:** Effect of the used device to three-class stress with context classification accuracy when heart activity and accelerometer data are used together.

Algorithm	Empatica E4	Samsung Gear S-S2	All Devices
PCA + LDA	88.88	72.60	59.12
PCA + SVM (rad)	92.06	78.60	76.91
kNN	87.30	85.30	87.30
Logistic Regression	90.47	83.30	65.25
Random Forest	90.40	**88.60**	**88.30**
Multilayer Perception	**95.23**	87.30	83.10

**Table 8:** Effect of the device used to three-class stress level classification accuracy when only heart activity signal is used (without context).

Algorithm	Empatica E4	Samsung Gear S-S2	All Devices
PCA + LDA	65.07	55.33	52.58
PCA + SVM (rad)	**90.40**	73.33	62.60
kNN	88.88	82.00	82.15
Logistic Regression	84.90	66.66	66.66
Random Forest	87.30	**84.67**	**82.62**
Multilayer Perception	88.88	78.00	71.36

**Table 9:** Classification accuracies vs. changing percentage based artifact detection and filtering rules.

Algorithm	10%	15%	20%	25%
PCA + LDA	64.28	62.38	59.62	63.80
PCA + SVM(rad)	80.95	78.57	77.00	79.52
kNN	87.61	86.66	87.32	**85.20**
Logistic Regression	73.80	61.90	66.25	66.19
Random Forest	**89.00**	**88.09**	**89.26**	82.60
Multilayer Perception	80.00	78.57	83.09	80.95

**Table 10:** Effect of the length of the aggregation window on classification accuracies.

Algorithm	Aggregation Window Size (s)			
	120	300	600	1200
PCA + LDA	59.62	62.24	54.14	63.02
PCA + SVM (radial)	76.99	77.94	77.27	**83.33**
kNN	87.32	83.30	**88.38**	85.41
Logistic Regression	65.25	69.60	72.22	76.16
Random Forest	**88.26**	86.76	87.87	84.14
Multilayer Perception	83.09	**86.76**	81.81	88.54

**Table 11:** Classification accuracies of Empatica E4 when removed inter-beat interval artifacts are replaced with interpolation vs. when they are removed.

Algorithm	Filtering	Interpolation
PCA + LDA	72.72	50.75
PCA + SVM	89.39	89.39
kNN	**95.45**	**97.72**
Logistic Regression	83.33	89.39
Random Forest	**95.45**	93.93
Multilayer Perception	89.39	95.45

**Table 12:** Classification accuracies of general and person-specific models.

Algorithm	General		Person Specific	
	HR + EDA + ACC-E4	HR + ACC-All	HR + EDA + ACC-E4	HR + ACC-All
PCA + LDA	82.35	59.12	95.83	87.60
PCA + SVM (radial)	82.35	76.99	93.75	85.98
kNN	80.39	87.30	95.83	89.91
Logistic Regression	90.19	65.25	95.83	90.17
Random Forest	86.27	**88.20**	**97.92**	90.17
MLP	**92.15**	83.20	95.83	**91.54**

**Table 13:** Classification accuracies comparison of subjective report and known context. On the left, known context information (Free,1; Lecture, 2; Contest, 3) was used as class labels. On the right, subjective ground truths are used as class labels.

Algorithm	Accuracy Wrt. Known Context		Accuracy Wrt. Subjective Ground Truth	
	HR + ACC-All	HR + EDA + ACC-E4	HR + ACC-All	HR + EDA + ACC-E4
PCA + LDA	59.12	82.35	54.46	50.98
PCA + SVM (radial)	76.99	82.35	69.01	72.55
kNN	87.30	80.39	85.44	**78.44**
Logistic Regression	65.25	90.19	57.27	78.43
Random Forest	**88.20**	86.27	**86.38**	76.47
MLP	83.20	**92.15**	80.28	68.62
